# Recurrent Duplication, Testis-Biased Expression, and Functional Diversification of *Esf2/ABT1* Family Genes in *Drosophila*

**DOI:** 10.3390/insects16090956

**Published:** 2025-09-11

**Authors:** Elizaveta D. Davydova, Alexei A. Kotov, Alina V. Chernizova, Ekaterina Yu. Yakovleva, Ludmila V. Olenina

**Affiliations:** 1 Laboratory of Functional Genomics, Koltzov Institute of Developmental Biology, Russian Academy of Sciences, 26 Vavilov Str., 119334 Moscow, Russia; elizaveta.dav@yandex.ru (E.D.D.); kotov_alexei@mail.ru (A.A.K.); e.u.yakovleva@gmail.com (E.Y.Y.); 2 Laboratory of Cellular Neurobiology of Learning, Institute of Higher Nervous Activity and Neurophysiology, Russian Academy of Sciences, 5A Butlerov Str., 117485 Moscow, Russia; alisach64@gmail.com

**Keywords:** gene duplication, sex-biased expression, *Drosophila*, piRNA cluster, ABT1-like domain, Intrinsically Disordered Regions

## Abstract

**Simple Summary:**

To investigate the evolutionary history of *Esf2/ABP1* gene duplicates in *Drosophila* species, we performed a search and subsequent characterization of *Esf2/ABP1* copies using comparative genomic analysis, phylogenetic analysis, functional domain analysis, and tissue-specific transcriptomic analysis. We determined that despite the single copy of the *Esf2/ABP1* gene per genome harbored in the majority of *Drosophila* species, recurrent gene duplication events occurred in four species of the *melanogaster* subgroup and independently in three species of the *suzukii* subgroup. DNA-mediated duplications took place on the X chromosome in close proximity to the parental copy or involved translocation and amplification of duplicated copies within the introns of unrelated genes or within heterochromatic piRNA cluster. We found that novel functions of duplicated copies emerged through various evolutionary processes, leading to the development of new adaptations, including testis-enriched expression for the beneficial increase in gene dosage and protein domain acquisition and diversification.

**Abstract:**

Gene duplications are considered to be the major evolutionary resource of novel functions. The gene family *Esf2/ABP1* is conserved in metazoan organisms from yeast to humans. Here we performed a search and characterization of *Esf2/ABP1* homologs in the *Drosophila* genus. Whereas in the majority of *Drosophila* species this gene family is represented by only a single gene, in the *melanogaster* and *suzukii* subgroups recurrent gene duplications arose, providing 47 homologous genes located on the X chromosome. To study the evolutionary history of duplicates, we performed phylogenetic, functional domain, and tissue-specific expression analyses. We revealed a male-specific and testis-biased transcription pattern of duplicated copies in *Drosophila melanogaster* and *Drosophila sechellia* compared to ubiquitous expression of the parental gene. The amplification of 21 repeated paralogs within the heterochromatic piRNA cluster resulted in the ovarian-specific transformation of these repeats into piRNAs in *D. melanogaster.* In three species of the *suzukii* subgroup, *Esf2/ABP1* genes evolved with domain diversification: in addition to RNA-binding ABT1-like domain preservation, all homologous proteins acquired expanded intrinsically disordered regions. By studying the duplicated copies of the *Esf2/ABP1* family in *Drosophila*, we offer insight into how novel gene functions emerge and are maintained, contributing to life’s diversity and complexity.

## 1. Introduction

Gene duplication followed by divergence is now considered one of the fundamental mechanisms driving the evolution of new functions in metazoan organisms. Duplicated genes provide substrates from which evolution can generate novel functions, and thus duplications contribute to processes of environmental adaptation, speciation, and increasing organismic complexity. Gene duplication can be raised in different ways, including tandem gene duplication, whole-genome and segmental chromosomal duplication, and also retroposition or transposition. Following gene duplication, a newly duplicated gene copy can realize different fates. Most often it is a pseudogenization with accumulation of degenerative changes, e.g., mutations, frameshifting deletions, or premature stop codons owing its functional redundancy [1,2]. However, another part of duplicated genes could acquire new functions and be retained in the genome.

According to the modern view, multiple processes can functionally preserve duplicated genes, including conservation, neofunctionalization, subfunctionalization, specialization, dosage benefit, duplication–degeneration–complementation, and others [3,4,5,6]. When increased gene dosage is beneficial, ancestral functions are conserved in both gene copies. During neofunctionalization, one gene copy carries out its ancestral functions, and the other one acquires a novel function and expression pattern [2,3,6]. A subfunctionalization process means that copies are mutated differently with a maintenance of ancestral gene functions by both genes [2,4]. Concerted action of subfunctionalization and neofunctionalization processes provides a specialization of gene copies that functionally evolved from each other and from the ancestral gene.

The gene family *Esf2/ABP1* includes genes that are phylogenetically conserved in metazoan organisms from yeast to humans. These genes encode the nuclear protein ABT1 (activator of basal transcription 1) and its yeast homolog, pre-rRNA-processing protein Esf2 (eighteen S factor 2). The molecular function of ABT1 is described as a TBP-binding regulator of basal transcription for PolII genes in mice [7]. In *Saccharomyces cerevisiae,* Esf2 is known as a nucleolar RNA-binding protein involved in pre-rRNA processing and the biogenesis of small ribosomal subunits [8]. The functions of Esf2 were found to be essential in yeast [7]. Despite the molecular functions of Esf2/ABP1 proteins in different metazoan organisms not being clearly understood yet, in *Drosophila melanogaster* the *Esf2/ABP1* family gene *CG32708* has been found to be essential for fly development and associated with the Notch signaling pathway and cell proliferation [9,10,11,12].

The *Drosophila* represents a clear and attractive model system to study the evolutionary forces acting on the evolutionary dynamics of gene copies [13,14,15,16,17,18]. The recent appearance of high-quality genome assemblies of *Drosophila* species, including challenging heterochromatin genomic regions with arranged genomic repeats [19,20,21], provides a valuable opportunity to understand genetic and evolutionary mechanisms that drive the maintenance and divergence of *Esf2/ABP1* family genes. In this study we report our initial functional and evolutionary characterization of *Esf2/ABP1* gene family duplications in genomes of *Drosophila*. We found that in the majority of *Drosophila* species, this gene family is represented by only a single gene maintaining original functions. However, in the *melanogaster* subgroup as well as in the *suzukii* subgroup, we identified recurrent gene duplications providing, in sum, at least 47 homologous genes expanding to distinct genomic locations on the X chromosome. Investigating the origin, divergence, phylogenetic relationships, and expression pattern of *Esf2/ABP1* genes, we revealed a sex-specific expression bias of duplicated copies in *Drosophila melanogaster* and *Drosophila sechellia* and functional diversification of homologs in the *suzukii* subgroup species, indicating evolutionary adaptation of newly raised gene copies.

## 2. Materials and Methods

### 2.1. Homolog Search and Identification

We performed a search of *Esf2/ABP1* homologs and duplicates in the *Drosophila* genus using the Ensemble Metazoa tool (https://metazoa.ensembl.org/ accessed on 2 April 2025) and *CG327708* gene as a query. The analysis of the identity of homologs was performed using the coding and protein sequences identified by Ensembl (https://ftp.ebi.ac.uk/ensemblgenomes/pub/release-60/metazoa/fasta/ accessed on 20 May 2025). Sequences were pairwise aligned using MUSCLE v3.8.1551 [22]. The percent identity was calculated as the number of matching bases/amino acids divided by the number of alignment columns, excluding gaps. The data were visualized as the heatmap using the Python 3.11 libraries, Matplotlib 3.10.5 and Seaborn 0.13.2. For microsynteny estimation we analyzed up to three upstream and three downstream surrounding genes for each location using NCBI Genome Data Viewer (https://www.ncbi.nlm.nih.gov/gdv?org accessed on 25 May 2025) and corresponding genome assemblies. Genes are considered to be in a syntenic location if they share more than 15% flanking homology.

### 2.2. Phylogenetic Analysis

For search *Esf2/ABP1* family homologous genes 23 whole genome assemblies of *Drosophila* species (NCBI RefSeq) were used with the BLAST +2.17.0 (blastn) algorithm and *CG327708* gene as a query. Corresponding genome assemblies are listed in Table S1. For multiple alignment we retrieved the CDS for homologous genes of closely related species with reference genome assemblies including *Drosophila simulans* Prin_Dsim_3.1 GCA_016746395.2, *Drosophila mauritiana* ASM438214v1 GCA_004382145.1, *Drosophila sechellia* ASM438219v2 GCA_004382195.2, *D. yakuba* Prin_Dyak_Tai18E2_2.1 GCA_016746365.2, and *Drosophila melanogaster* Release 6 plus ISO1 MT GCA_000001215.4 from the NCBI genome database. The nucleotide sequence alignment was carried out using MEGA 12 software [23] via the MUSCLE method [22] for codons. The alignments required minimal manual editing and G-blocks 0.91.1 [24] treatment with following parameters: data type: codon; max number of contiguous non-conserved positions: 8; min length of a block: 10; allowed gap position: with half. Phylogenetic tree constructions were performed using the MEGA 12 software. The Maximum Likelihood method was used with the Tamura–Nei model of nucleotide substitutions [25] for the tree generation with highest log likelihood (−1933.81) and *Drosophila yakuba* sequence of *LOC6525244* gene as the outgroup. The percentage of replicate trees in which the associated taxa clustered together (500 replicates) is shown below the branches. Gamma distribution was used to model evolutionary rate differences across five categories (+G, parameter = 1.9616), with 0.00% of sites deemed evolutionarily invariant (+I). The partial deletion option was applied to eliminate all positions with less than 95% site coverage resulting in a final data set comprising 351 positions. The analytical procedure encompassed 42 coding nucleotide sequences using first, second, third, and non-coding positions. Evolutionary analyses were conducted in MEGA 12 [23] utilizing up to four parallel computing threads.

### 2.3. Z-Test of Neutrality

To estimate the type of selection for the *Esf2/ABP1* family genes in *D. melanogaster,* we applied the codon-based test of neutrality, the Z-test, using MEGA 11 software [23,26]. Analyses were conducted using the Nei–Gojobori method [27]. This analysis involved two aligned nucleotide sequences. All ambiguous positions were removed for each sequence pair (pairwise deletion option). The test statistic (*dN* − *dS*) is represented as a Z-score, where *dS* and *dN* are the numbers of synonymous and nonsynonymous substitutions per site, respectively.

### 2.4. Search for Protein Domains

For the determination of known protein domains of *Esf2/ABP1* family proteins, NCBI’s Conserved Domain Database (CDD) tool (https://www.ncbi.nlm.nih.gov/Structure/cdd/wrpsb.cgi accessed on 24 June 2025) [28] was used. The search was performed against database CDD v3.21 with an expected value threshold of 0.01. Only specific hits were considered. Intrinsically disordered regions (IDRs) were predicted with the Critical Assessment of Intrinsic Protein Disorder (CAID) Prediction Portal (https://caid.idpcentral.org/ accessed on 26 June 2025) [29] with a default threshold of 0.5. The overall disorder scores for the proteins expressed as 50% of IDRs in the whole protein length were considered as significant.

### 2.5. Fly Stocks

To analyze the transcript level in different fly tissues and copy number estimation we used flies of wild-type strains of *D. melanogaster Batumi L*, *Harwich*, *GH10*, and *GH16*. *Batumi L* and *Harwich* strains were obtained from the collection of laboratory and natural strains of *Drosophila* of Institute of Developmental Biology, RAS, Russia. Strains from African population (Ghana, Africa), *GH10* and *GH16* [30], were obtained from the *Drosophila* collection of Institute of Cytology and Genetics, SB RAS, Russia. *D. sechellia* stock was obtained from the Gif-sur-Yvette CNRS Center collection, France. All flies were reared at 23 °C on a standard medium. The flies were subjected to a laboratory light–dark cycle of 12:12 h.

### 2.6. RT-qPCR Analysis

For RT-qPCR analysis, total RNA was isolated from gonads, heads, and carcasses of 3–5-day adult male and female flies with TRIzol Reagent (Invitrogen, Waltham, MA, USA). A DNA-free Kit DNA Treatment & Removal (Invitrogen) kit was used for the removal of genomic DNA from preparations. Reverse transcription was performed using Mint kit (Eurogen, Moscow, Russia) and Oligo(dT) primers. cDNA samples were analyzed by real-time quantitative PCR using the incorporation of SYTO13 (Invitrogen). All experiments were performed with at least three independent RNA samples; each sample was analyzed in triplicate. Fold change ratios of the average expression level were calculated. We used *rp49* (*rpL32*) as a loading control. The following primers were used: *rp49* fw 5′-ATGACCATCCGCCCAGCATAC-3′, rev 5′-GCTTAGCATATCGATCCGACTGG-3′; *cuckoo* fw 5′-CATGGAAGTAGAGGAGGCTGAG-3′, rev 5′-GGTATGTTGGATATGTAGATGATCCC-3′; *chaffinch* (shared) fw 5′-AAATGACGATAAAAAGGAGCTGG-3′, rev 5′-TGTCCTTGGGTATGTTGGATATTA-3′; *brambling* fw 5′-CTGAGATGCCGTTCTCTTATAAGACG-3′, rev 5′-CAAGGTCATGTGCTTGGGTAGGT-3′; *woodpecker* (shared) fw 5′-GGAGGAGATTGAGACCTCGG-3′, rev 5′-GTGCTTGGGTATGTTGGATATGT-3′; *D. sechellia* parental copy *LOC6619759* fw 5′-GGTGCACAAGCAACGCCTA-3′, rev 5′-CAGAGCCTTTTCCGCTTTCTT-3′; *D. sechellia* duplicated copies of cluster 1 (*LOC6621826, LOC116802136, LOC116802134, LOC116802120, LOC6619761*) (shared) fw 5′-CTGAGAAACCCGAGAAGATGCA-3′, rev 5′-TTAACAGCTCTTTCGGCTGCAC-3′; *D. sechellia* cluster 2 duplicates (*LOC6618463, LOC116801944, LOC6618464, LOC6618462*) (shared) fw 5′-AACGAACAAAAATGAATTCTGAGCC-3′, rev 5′-CATTCGCAGACTCACCTTCCTC-3′.

### 2.7. Copy Number Estimation of Woodpecker Genes

To estimate the number of *woodpecker* gene copies in the *D. melanogaster* genome, quantitative PCR was performed with genomic DNA of *Batumi L*, *Harwich*, *GH10*, and *GH16* wild-type strains to determine the concentration level of amplification products of each of the genes of the family. We used highly specific *woodpecker* primers (see above), the efficiency of which was previously tested and compared with the efficiency of *rp49* primers (normalization control). Measurements for each strain were carried out in three replicates. We used new genomic assemblies of isofemale *iso-1*, *A4*, and *A3* strains (available in the NCBI genome database as GCA_042606445.1, GCA_042606465.1, and GCA_042606455.1) [31] to determine the number of *woodpecker* genes per genome with the aid BLAST.

### 2.8. Transcriptomic Analyses of Esf2/ABP1 Family Genes Using Deep Sequencing Library Data

PolyA + RNA-seq libraries from *w*^1118^ strain of *D. melanogaster* embryos, third instar larval gonads and sex-separated adult tissues/body parts and gonads of *w*^1118^ and *aub* mutant flies [32,33,34,35,36,37] (Table S2) were used for expression analysis (from duplicate to quadruplicate of libraries for tissue). The libraries were pseudo-aligned using Kallisto v.0.50.0 with the following parameters—single −l 100 −s 0.05 [38]. The *D. melanogaster* transcriptome set obtained from the NCBI was used as a reference (GCF_000001215.4). Transcript abundances were used to assess expression in terms of transcripts per million (tpm). The data were visualized using the Python libraries, Pandas, Matplotlib, and Seaborn.

### 2.9. Bioinformatics Analysis of piRNAs

For piRNA distribution analysis in the *AT-chX* cluster, small RNA libraries from *Oregon R* ovaries [39] and *w*^1118^ (*yw*) testes [40] of *D. melanogaster* were used (Table S2). To assess the effect of the *aub* mutation on the production of piRNA to *woodpecker* repeats in the gonads of *D. melanogaster*, libraries of short RNAs from the ovaries of *aub^HN^*^2^*/aub^QC^*^42^ females and heterozygous control were used [41] (Table S2). Adapter sequences from small RNA libraries were removed by cutadapt 2.8. The FastQC tool was used for quality control of the sequenced libraries. Reads were filtered by length (23–30) and quality (Phred quality score > 30) using fastq-filter (https://github.com/LUMC/fastq-filter?tab=readme-ovfile#readme, accessed on 4 April 2025). rRNA, snRNA, snoRNA, microRNA, and tRNA were filtered off from the libraries. To analyze the reads generated from the *AT-chX* and *woodpecker* repeat containing piRNA clusters, locally unique reads (reads could only be mapped to genomic region with coordinates chrX:21631000–22440000 containing *AT-chX* repeats, *woodpecker* repeats, and a neighboring *flamenco* piRNA cluster) were used. For this, libraries were mapped to the Release 6 plus ISO1 MT genome assembly without contigs and chrX:21631000–22440000 region. Unmapped reads were remapped to the chrX:21631000–22440000 region using bowtie with −n 1 −l 30 parameters. The mapped reads were visualized using Integrative Genomics Viewer (IGV) (https://www.broadinstitute.org/scientific-community/software/integrative-genomics-viewer, accessed on 8 April 2025). To evaluate the potential silencing of *Esf2/ABP1* family genes by piRNAs, the *Oregon R* ovarian library was independently mapped to gene sequences using bowtie (−n 2). Small RNA libraries from flies with *aub* mutation after preprocessing (see above) were mapped to the *woodpecker* repeats sequence using bbmap.sh (minid = 0.95). The mapped reads were counted and normalized. Ping-pong signatures for sense and antisense piRNA pairs were calculated using the signature.py script [42].

## 3. Results

### 3.1. Survey of Recurrent Duplications of Esf2/ABP1 Family Genes in Drosophila

**Figure 1:**
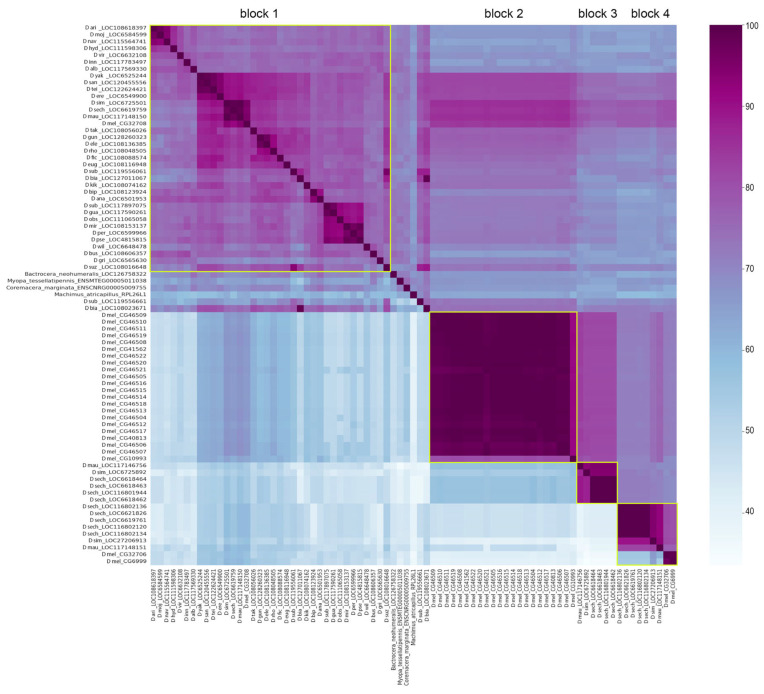
Heatmap profile of pairwise alignment by MUSCLE v3.8.1551 of sequences of *Esf2/ABP1* family homologs from species across the genus *Drosophila* (see Figure S1 for the phylogenetic tree). Data above the diagonal line show the nucleotide alignment identity of the CDS sequences. Data below the diagonal line show the protein alignment identity of the amino acid sequences. Identity percent for each pairwise alignment was calculated as the ratio of the number of matching bases/amino acids to the number of alignment columns, excluding gaps. *Machimus atricapillis* gene *RPL26L1* was used as an unrelated control. Several data blocks are highlighted with rectangular yellow frames and designated on the top. The heatmap scale is represented on the right.

Multiple animal species represent genes belonging to the *Esf2/ABP1* family in their genomes as a single homologous copy per genome. Using the Ensemble Metazoa tool (https://metazoa.ensembl.org/ accessed on 2 April 2025), we identified a single *Esf2/ABP1* gene per genome for the majority of *Drosophila* genus species; however, we revealed gene duplication events for several species of the *melanogaster* group (Figure S1). Among them there are four species of the *melanogaster* subgroup, *Drosophila melanogaster*, *Drosophila simulans*, *Drosophila mauritiana*, and *Drosophila sechellia*, and species of the *suzukii* subgroup, *Drosophila biarmipes*, and *Drosophila subpulchrella*. Some of the *Esf2/ABP1* gene duplications for *D. melanogaster*, *D. simulans*, and *D. mauritiana* have been found earlier [43]. With pairwise sequence alignments between homologs, we revealed that high sequence homology is preserved across the genus *Drosophila* both for nucleotide sequences of their CDSs and for translated amino acid sequences (Figure 1).

**Figure 2:**
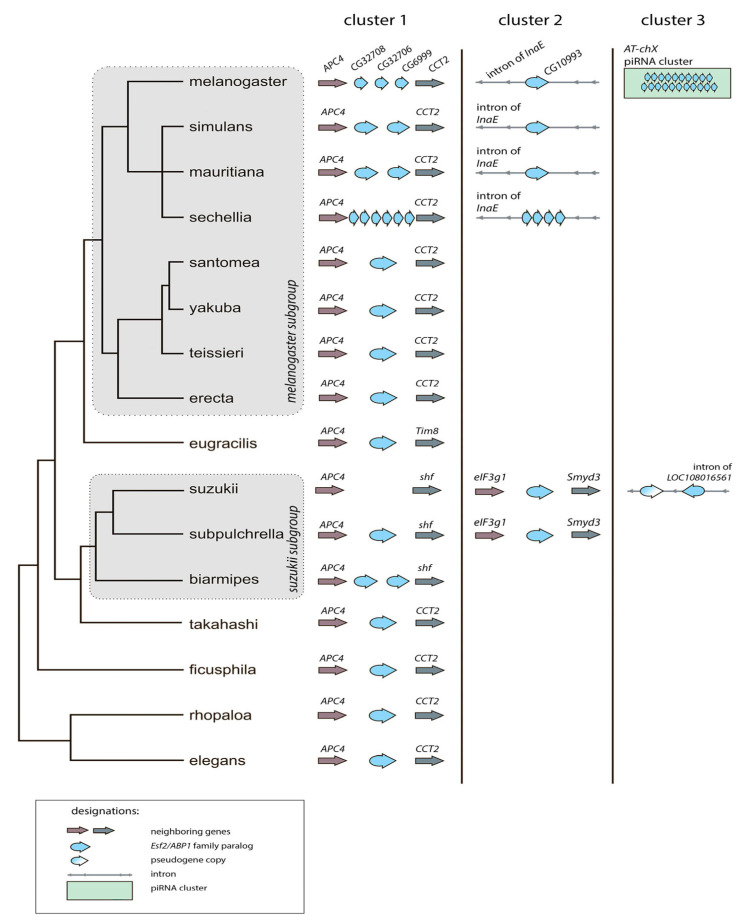
Survey of duplication events and synteny analysis of *Esf2/ABP1* homologs. The presence, copy number, and absence of homologous genes across the phylogeny of the *melanogaster* group are represented. The number of blue figures in each location indicates the copy number of *Esf2/ABP1* homologs. Synteny analysis showed that a single *Esf2/ABP1* copy is located on the X chromosome, mainly flanked by *APC4* and *CCT2* genes (see Table S1 for additional info). We found that four species of the *melanogaster* subgroup, *D. melanogaster*, *D. simulans*, *D. mauritiana*, and *D. sechellia*, and three species of the *suzukii* subgroup, *D. suzukii*, *D. biarmipes*, and *D. subpulchrella*, are subjected to gene duplication events. We subdivided the genomic location of duplicated copies into three clusters for each subgroup as indicated on the top of the figure. Note that, except for cluster 1, the genomic locations of the clusters do not coincide for these subgroups except for their distribution on the X chromosome.

To characterize *Drosophila Esf2/ABP1* homologs in detail, we focused on 16 species of the *melanogaster* group of the subgenus *Sophophora* (∼25 MIA divergence between species). Through the leverage of high-quality *Drosophila* genome assemblies (https://www.ncbi.nlm.nih.gov/datasets/genome/, accessed on 23 May 2025), we used blastn-based approach to identify *Esf2/ABP1* orthologs and paralogs across the *Sophophora* species and subjected all the found genes to the synteny analysis (Figure 2, Table S1). Our analysis revealed that duplicated gene copies in *D. melanogaster* and species of the *simulans* clade are clustered at three distinct genomic locations on the X chromosome. The first region (cluster 1) is located within a gene-rich euchromatin environment between *APC4* (*Anaphase Promoting Complex subunit 4)* and *CCT2* (*Chaperonin containing TCP1 subunit 2*) genes (Figure 2). This region contains at least one copy of the *Esf2/ABP1* family genes in most analyzed genomes. This region of *D. melanogaster* (cytolocation 8C7) contains three paralogous *Esf2/ABP1* genes, *CG32708*, *CG32706*, and *CG6999*, as has been described earlier [43]. Among them, the upstream *CG32708* gene has a high nucleotide identity with homologous genes of other *Drosophila* species (Figure 1 block 1). Orthologous genes of the *melanogaster* group that are predominantly located between *APC4* and *CCT2* genes on the X chromosome have nucleotide identity to *CG32708* gene for their CDSs in the range of 75.2–95.4% (Figure 1 and Figure 2, Table S1). *CG32708* is characterized by ubiquitous expression in different fly tissues (according to FlyAtlas2 data) and is shown as the essential for developmental and proliferation processes [9,10,11,12]. Thus, the most parsimonious explanation is that *CG32708* is the progenitor gene copy maintaining original functions in *D*. *melanogaster*. Taking all this into account, we assume that *CG32708* and its orthologs in this location appear to be parental copies from which the following paralogous genes originated in the *melanogaster* group. We found the parental copy and one duplicated copy in this genomic location in *D. simulans* and *D. mauritiana* genomes. However, in *D. sechellia* genome we observed in this region the parental copy with the highest homology 94.7% to *CG32708* and downstream five almost identical tandem gene duplications with only 66.8–67.3% homology to *CG32708* (Figure 1 block 4 and Figure 2, Table S1). Considering that *D. melanogaster* has split from an ancient precursor with the *simulans* clade about 4.3–6.5 MYA [44,45,46,47,48], we proposed that first duplication event in this genomic region occurred before *D. melanogaster* divergence from sibling species.

The second X chromosome region (cluster 2) containing *Esf2/ABP1* copies is the intron of the *InaE* (*inactivation no afterpotential E*) gene of the *melanogaster* subgroup (cytolocation 12C5 for *D. melanogaster*). Whereas, only single *Esf2/ABP1* orthologs were found for *D. melanogaster*, *D. simulans*, and *D. mauritiana* species in this genomic location; for *D. sechellia*, we observed four highly identical tandem duplicated paralogs (Figure 1 block 3 and Figure 2, Table S1). The strong syntenic location of these genes also indicates that the initial gene insertion into the *InaE* intron and subsequent fixation occurred before the splitting of *D. melanogaster* from the species of the *simulans* clade.

**Figure 3:**
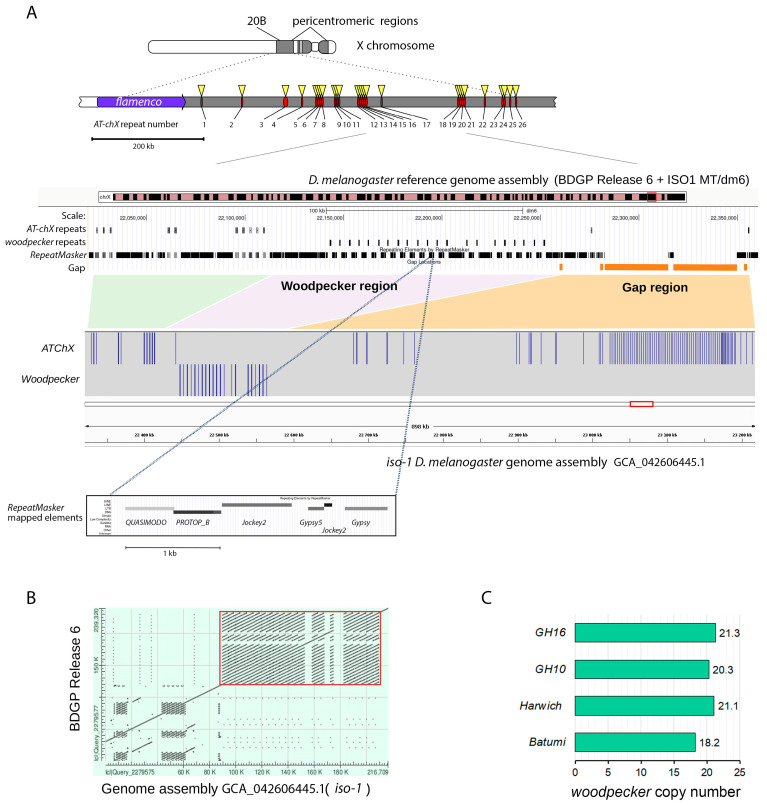
(**A**) Distribution of *Esf2/ABP1* paralog repeats (*woodpeckers*) within the *AT-chX* piRNA cluster on the pericentromeric region of the *D. melanogaster* X chromosome. On the top: The scheme of the germline-specific *AT-chX* piRNA cluster is shown according to [40]. In the middle: *Woodpecker* repeat visualization in the *D. melanogaster reference* genome assembly Release 6 plus ISO1 MT. At least 21 *woodpecker* repeats are located between *AT-chX 17* and *AT-chX* 18 repeats within a 109.58 kb region in cytolocation 20B. The view encompassing the inner part of the *AT-chX* piRNA cluster is shown. Several *AT-chX* repeats upstream and downstream of the *woodpecker* repeat-containing region accompanied by *RepeatMasker* mapping are indicated in the browser view. On the bottom: We verified the structure of the analyzed locus in the genome assembly of the *iso-1* strain. In this new assembly [31], the gaps present in the reference genome assembly (orange bars) were filled. The filled gaps contain mainly transposon fragments as well as previously unannotated copies of *AT-chX* repeats but do not contain sequences homologous to *woodpecker* repeats. The region containing 21 *woodpecker* genes has the same structure in both genome assemblies. Using the *RepeatMasker* tool, we identified a repeating pattern of fused transposon fragments that separate individual *woodpecker* copies from each other (on the bottom square, one magnified unit of fused transposon fragments is presented). (**B**) The dot matrix based upon the BLAST results shows *woodpecker* region similarity between two compared genomic assemblies. The *woodpecker* region (red square) has the identical structure in both genome assemblies. (**C**) The *woodpecker* copy number estimation in four wild-type *D. melanogaster* strains (see Section 2 for details).

We uncovered the third region (cluster 3) only in *D. melanogaster* genome, containing at least 21 *Esf2/ABP1* gene copies (Figure 2 and Figure 3A, Table S1). This region is located in pericentromeric heterochromatin of the X chromosome (cytolocation 20B). It has been identified earlier as the expanded germline-specific piRNA cluster containing *AT-chX* repeats [35,40]. piRNA clusters are specialized genomic regions responsible for producing the majority of small non-coding piRNAs in germinal tissues [41,49]. The *Esf2/ABP1* paralogs in this region of *D. melanogaster* are arranged tandemly, with blocks of fused-together transposon fragments of repeating patterns placed between neighboring paralogous copies (Figure 3A). Despite their location within the active piRNA cluster, these duplicated copies are annotated as genes in the reference genome assembly; each copy contains one intron and possesses the highest homology among themselves (more than 99%) (Figure 1 block 2). We designated these repeats as *woodpeckers* with corresponding numbers (*woodpecker 1, woodpecker 2, …, woodpecker 21*) (Figure 3A). We also analyzed the number of *woodpecker* copies in new genomic assemblies of *D. melanogaster iso-1*, *A4*, and *A3* isofemale strains with accurately assembled pericentromeric heterochromatin [31] and found 21, 25, and 24 genes, respectively. We verified the structure of the analyzed locus on the genomic assembly of *iso-1* strain (Figure 3A). The dot matrix (Figure 3B) based upon the BLAST results shows *woodpecker* region similarity between two compared genomic assemblies. The region containing 21 *woodpecker* genes has the same structure in both genome assemblies. We estimated *woodpecker* gene copy number in four wild-type *D. melanogaster* strains (*Batumi, Harwitch, GH10*, and *GH16*) using quantitative PCR of genomic DNA (Figure 3C). There are 18–21 *woodpecker* copies in the analyzed genomes. The absence of these repeats in the genomes of other *Drosophila* species, including closely related ones of the *simulans* clade, indicates their recent arising, fixation, and maintenance only in *D. melanogaster.*

According to our analysis, duplication events for the *Esf2/ABP1* gene family have occurred also in the *suzukii* subgroup. Both *D. biarmipes* and *D. subpulchrella* genomes contain parental *Esf2/ABP1* copies with 76.2% and 81.5% nucleotide identity to *CG32708*, respectively, in the genomic location flanked upstream by *APC4* gene, and, in addition to that, *D. biarmipes* has a second paralog in this location (Figure S1 and Figure 2 cluster 1, Table S1). *D. subpulchrella* genome also contains a single *Esf2/ABP1* copy in a non-syntenic region of the X chromosome, between *eIF3g1* and *Smyd3* genes (Figure 2 cluster 2, Table S1). Our search revealed that in their closely related species *D. suzukii*, the parental *Esf2/ABP1* gene near *APC4* gene has been apparently lost, but instead we identified one copy between *eIF3g1* and *Smyd3* genes, and additionally two duplicated copies, a gene and a pseudogene, in the intron of X-linked *LOC108016561* gene encoding the ortholog of *D. melanogaster CG42594* gene (Figure 2 clusters 2 and 3, Table S1).

The pattern of *Esf2/ABP1* homolog distribution in the *suzukii* subgroup species indicates that gene arising and loss is a rather independent process compared with species of the *melanogaster* subgroup. However, for both subgroups we observed that the parental copy and all duplicated copies are located exclusively on the X chromosome. It should be noted that the Y chromosome is not consistently represented in the available genome assemblies; for this reason, we were unable to systematically search of gene duplication on the Y chromosome, except for *D. melanogaster* and *D. suzukii* assemblies (see Table S1). However, for the *simulans* clade, we used gene annotation data of recently improved Y chromosome long-read assemblies [50] and also did not observe Y-linked *Esf2/ABP1* homologs. Beyond the *melanogaster* group, we did not detect *Esf2/ABP1* duplications in the *obscura*, *virilis,* and *repleta* groups considering only RefSeq annotated genome assemblies (data are shown in Table S1). Whereas in the *obscura* group we observed a partial synteny of the gene location; in the species of the *virilis* group of the distinct subgenus *Drosophila*, *Drosophila virilis,* the single *Esf2/ABP1* copy was found on the X chromosome at a non-syntenic location (Table S1). This suggests that the ancestral *Esf2/ABP1* gene changed its location after the divergence about 40–60 MYA [45,46] from a most recent common ancestor of this species and *D. melanogaster*. Taken together, we revealed several gene duplication events for this gene family in *Drosophila*, with the parental copy located nearby *APC4* gene being the *Esf2/ABP1* gene that remains a single-copy gene per genome across the majority of the *melanogaster* group species.

### 3.2. Phylogenetic Analysis for Homologous Genes of Melanogaster Subgroup

**Figure 4:**
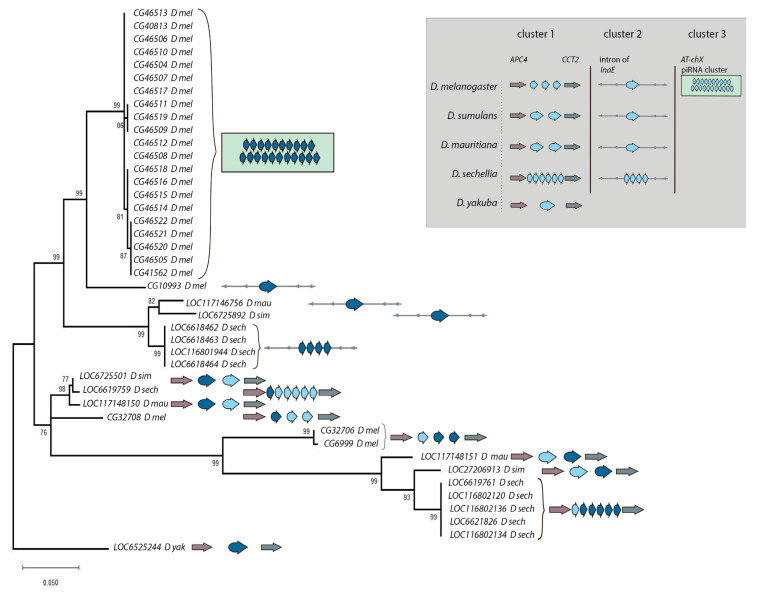
Maximum Likelihood phylogenetic tree for resolving the evolutionary relationship of *Esf2/ABP1* homologs in *D. melanogaster* and sibling species of the *simulans* clade with the highest log likelihood (−1933.81). Phylogenetic tree construction was performed using multiple nucleotide alignment of coding gene regions. The percentage of replicate trees in which the associated taxa clustered together (500 replicates) is shown near the branches. For the outgroup, *LOC6525244* gene of *Drosophila yakuba* was used. For all genes, their genomic locations are indicated by the icons according to Figure 2. Deeply colored blue figures on the icons mark genes corresponding to certain nodes. In the gray box on the top right, the common scheme of duplicated copy distribution in the analyzed genomes is represented according to Figure 2.

To evaluate the evolutionary relationship between homologs, we performed the collecting, multiple alignment, and phylogenetic analysis of coding sequences for all *Esf2/ABP1* family genes of *D. melanogaster, D. simulans, D. mauritiana,* and *D. sechellia* species. For generation of the phylogenetic tree, we used the Maximum Likelihood method and the Tamura–Nei model [25] with the orthologous *D. yakuba* gene *LOC6525244* as the outgroup (Figure 4). We have built the tree with the highest log likelihood (−1933.81) (Figure 4). Based both on tree characteristics and synteny analysis (Figure 2 and Figure 4), we proposed that duplication events of *Esf2/ABP1* genes in cluster 1 and cluster 2 regions of the X chromosome have occurred before the splitting of *D. melanogaster* from common ancestor species with the *simulans* clade. Orthologous genes from cluster 2 (embedded in the intron of *InaE* gene in all four analyzed genomes) turn out to be in two monophyletic groups: one group contains all genes from cluster 2 of the *simulans* clade species, whereas another group includes the homologous *CG10993* gene from cluster 2 and all 21 tandem *woodpecker* genes of cluster 3 of *D. melanogaster* (Figure 4). The close location of the branches of the last group on the tree indicates a potential origin of cluster 3 duplications from the single copy *CG10993* after speciation of *D. melanogaster.*

We evidently revealed a low level of divergence between the first upstream orthologous parental copies from cluster 1 for all four analyzed species. This information is consistent with our analysis of nucleotide and amino acid identity (Figure 1 block 1 and Figure 2, Table S1). The branch distribution for the following duplicated genes from cluster 1 inferred from the tree topology confirms high evolutionary rates for these copies; moreover, most divergence events appeared to occur before the splitting of the species. Thus, duplicated copies from cluster 1 have diverged considerably from their parental copies; however, within individual species, *D. melanogaster* and *D. seichellia*, all duplicated paralogs in cluster 1 are found to be extremely close to each other (Figure 1 block 4 and Figure 4). This branch forms a monophyletic group, indicating that the generation of the first duplicated copy occurred in a common ancestor of these species, whereas second gene duplications in these regions in *D. melanogaster* and *D. sechellia* appear to emerge from the first duplicated copies after the splitting of the species.

While most *Drosophila* species harbor a single *Esf2/ABP1* gene copy, the *D. melanogaster* genome contains multiple paralogs according to our and previously published data [43]. To facilitate subsequent analysis, we introduced new designations for them (Table 1). We defined the parental paralog *CG32708* as *cuckoo* because its daughter copies have been scattered at different locations of the X chromosome. Both duplicated copies in cluster 1, *CG23706* and *CG6999*, were named as *chaffinch 1* and *chaffinch 2*, correspondingly. We defined the single paralog *CG10993* in cluster 2 as *brambling*. Tandem amplified copies in cluster 3 were named as *woodpeckers* (*woodpecker 1, woodpecker 2, …, woodpecker 21*), as mentioned above.

To detect signatures of sequence evolution in *Esf2/ABP1* homologs of *D. melanogaster,* we performed the codon-based Z-test that estimates the ratios of synonymous and nonsynonymous divergence between analyzed genes and close orthologs of *D. simulans* (Table S3). We found that purifying selection maintains the parental copy, *cuckoo* (*CG32708*). In relation to other duplicated copies in all three genomic locations, we did not find significant deviations from neutral evolution.

### 3.3. Protein Domain Search in Esf2/ABP1 Proteins

**Figure 5:**
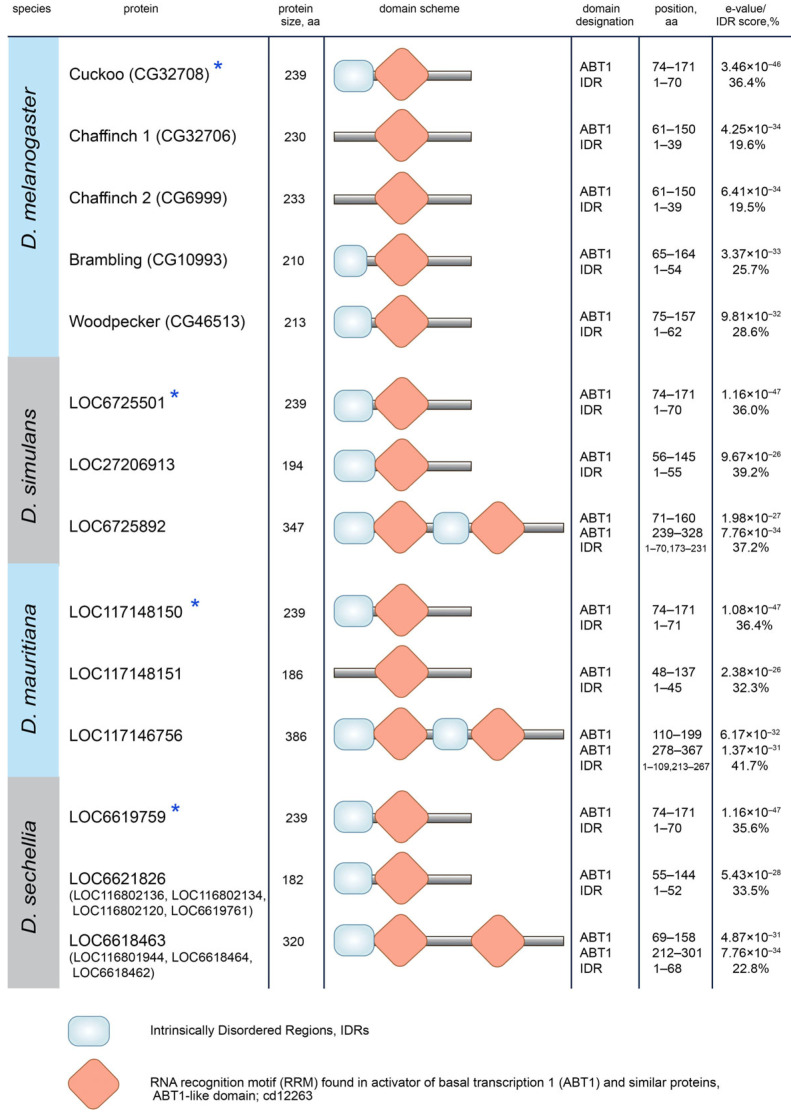
Protein domain determination for Esf2/ABP1 proteins of the *melanogaster* subgroup species. Blue asterisks mark proteins encoded by the parental gene copy in each species. All Esf2/ABP1 homologs, as predicted, retain the RRM ABT1-like domain with high e-value (last column). The overall disorder scores for the proteins are indicated as percentages in the last column. Only predicted Intrinsically Disordered Regions (IDRs) more than 50 aa in length are specified on the scheme. Note that we provided domain data for only Woodpecker 11 protein, CG46513, but the same properties were found for the other 20 Woodpecker proteins.

To detect potential innovations and functional sequence divergence in duplicated copies, we performed a search and comparison of known protein domains across Esf2/ABP1 homologs. Domain prediction for Esf2/ABP1 proteins of the *melanogaster* subgroup revealed that they shared with high e-value the RRM (RNA recognition motif) ABT1-like domain (domain found in activator of basal transcription 1) (Figure 5 and Figure S2) that has been firstly described for the Esf2/ABP1 family [7]. The most striking divergence occurred within the species of the *simulans* clade with duplicated copies embedded in the intron of *InaE* gene (cluster 2). The insertion of the duplicated copy into the intron of the unrelated gene was supplemented by the duplication of the extended internal fragment, providing a second RRM ABT1-like domain in these proteins (Figure 5 and Figure S2). We did not observe an intragenic duplication for the orthologous Brambling (CG10993) protein of *D. melanogaster,* indicating that the emergence and fixation of this whole-domain duplication occurred after the splitting of *D. melanogaster* from their common ancestor. All proteins analyzed also contain predicted Intrinsically Disordered Regions (IDRs) (with length from 39 aa to 109 aa); however, the overall disorder score for these proteins does not reach a high level (less than 50% of total length, from 19.5% to 41.7%) (Figure 5).

**Figure 6:**
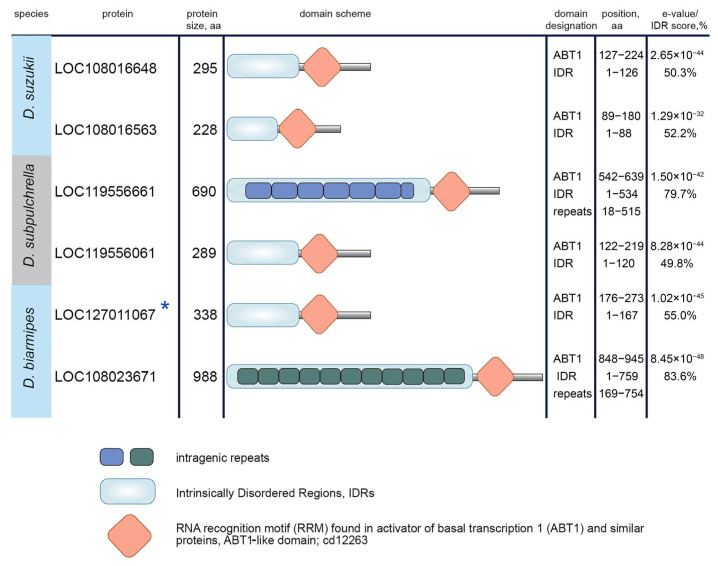
Protein domain determination for Esf2/ABP1 homologous proteins of the *suzukii* subgroup. A blue asterisk marks the protein encoded by the parental gene copy. All Esf2/ABP1 homologs retain the RRM ABT1-like domain. In addition, all proteins contain extended Intrinsically Disordered Regions (IDRs) at the amino terminus as indicated on the scheme. The overall disorder scores for the whole proteins are indicated as percentages in the last column. We did not specify predicted continuous IDRs less than 50 aa on the scheme. We identified multiple intragenic duplications for LOC119556661 of *D. subpulchrella* (6.5 repeats of 83 aa) and LOC108023671 of *D. biarmipes* (11 repeats of 54 aa) that significantly impact IDR expansion in these proteins.

Protein domain determination for Esf2/ABP1 proteins of the *suzukii* subgroup revealed that all Esf2/ABP1 homologs retain the RRM ABT1-like domain near their C-termini (Figure 6 and Figure S2). In addition, they contain extended continuous (in the range of 88 aa to 759 aa) IDRs at their N-termini (Figure 6). All of them have the overall disorder score equal to or exceeding 50% of the total length. Two proteins, LOC119556661 of *D. subpulchrella* and LOC108023671 of *D. biarmipes,* achieved a strongly increased relative size (up to 80% of the whole length) of their continuous IDRs due to multiple intragenic tandem duplications in these regions (Figure 6 and Figure S3). Thus, the divergence pattern of duplicated *Esf2/ABP1* genes in the *suzukii* subgroup is significantly different from that of the *melanogaster* subgroup duplicated orthologs. A gain of IDRs for duplicated copies is currently considered as a mechanism of the acquisition of new interaction partners and can reflect a putative new functional role [51,52].

### 3.4. Diversification of the Expression Pattern of Esf2/ABP1 Paralogs in D. melanogaster and D. sechellia

**Figure 7:**
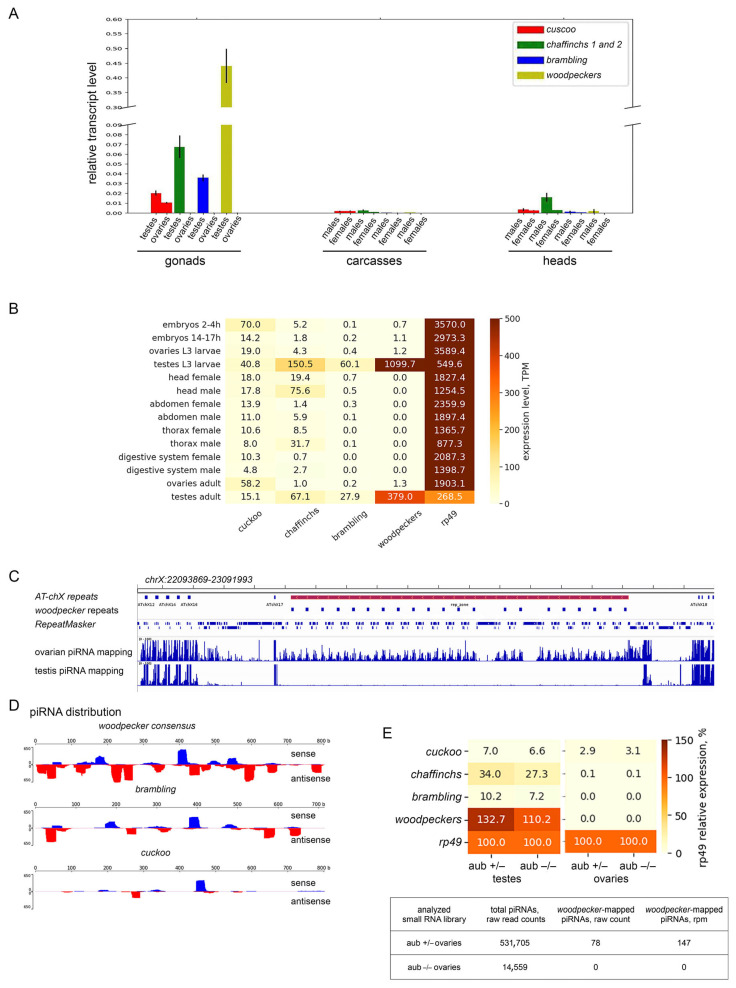
(**A**) RT-qPCR analysis of transcript levels of *Esf2/ABP1* family paralog in the gonads, carcasses, and heads of adult male and female flies of *w*^1118^ strain of *D. melanogaster*. The aggregated expression levels present for *chaffinch* and *woodpecker* genes. The expression levels of mRNAs were normalized to *rp49* transcripts. Error bars represent standard errors of the mean. (**B**) Expression analysis of *Esf2/ABP1* family genes from *w*^1118^ *D. melanogaster* in PolyA + RNA-seq libraries of embryos, third instar larval gonads, and adult tissues/body parts for each sex. The heatmap shows the expression of *cuckoo*, *brambling*, the collective gene expression of *chaffinch 1* and *chaffinch 2* genes, and *woodpecker* genes. *RpL32* (*rp49*) was used as the endogenous control (last column). Data are represented in TPM (transcripts per million). (**C**) Distribution of “locally unique” reads mapped to genomic region containing *woodpecker* repeats. The inner fragment of the *AT-chX* piRNA cluster encompassing *woodpecker* repeats (marked as rep_zone on the top (red bar)) is visualized using the Integrative Genomics Viewer (IGV). On the bottom: piRNAs map on the *woodpecker* repeat-containing region only in wild-type *D. melanogaster* ovaries but not in testes. The *y*-axis at the left represents read coverage of mapped piRNAs (number of reads per nucleotide position). (**D**) piRNA distribution across *woodpecker* consensus, *cuckoo,* and *brambling* coding sequences was performed, permitting 0–2 mismatches. The *y*-axis shows read coverage (read number per nucleotide position). The sense read density is shown in blue; the antisense read density is shown in red (in relation to *woodpecker*, *cuckoo*, and *brambling* transcripts). (**E**) Comparative analysis of differential expression of *Esf2/ABP1* family genes in gonads of *D. melanogaster* of piRNA mutants (*aub* −*/*−) and their heterozygous siblings (*aub +/*−) using RNA-seq library data. The expression levels were normalized to *rp49* transcripts taken as 100%. Up-regulation of *Esf2/ABP1* family genes in the case of disruption of the piRNA pathway is not observed. In the bottom table the results of analysis of ovarian *aub* +/− and *aub* −/− small RNA libraries are presented. In the ovaries of *aub* mutants, the total amount of piRNAs drops more than 35-fold, while ***woodpecker***-mapped piRNAs are not produced.

Using RT-qPCR and RNAseq data (Figure 7A,B), we revealed a sex-dimorphic pattern of *Esf2/ABP1* paralog expression in selected tissues of *D. melanogaster*. For differential expression analysis we used PolyA + RNAseq libraries of embryos, third instar larval gonads, and adult tissues/body parts for each sex. All *Esf2/ABP1* family genes are transcribed in the testes, with the highest transcript level found for the sum of *woodpecker* genes. In the ovaries we observed a noticeable level of transcripts only for *cuckoo* (*CG32708*), whereas other paralogs were not expressed. Parental gene *cuckoo* was found to be expressed ubiquitously in all selected tissues (Figure 7B) with the maximal transcript level in 2–4 h embryos, larval testes, and adult ovaries. Duplicated copies from cluster 1, *chaffinch 1 (CG32706)*, and *chaffinch 2 (CG6999)*, taken together, show maximal transcript level in larval and adult testes, heads, and thoraxes of adult males, demonstrating strong male-biased expression divergence from the parental gene. *Brambling* and *woodpeckers,* duplicated genes from clusters 2 and 3, correspondingly, exhibit a pronounced testis-specific expression pattern, likely associated with beneficial male germline activities (Figure 7B). Our RT-qPCR analysis of gonads, carcasses, and heads of adult flies of the wild-type *Batumi* strain of *D. melanogaster* generally confirmed the results obtained using RNAseq expression analysis (Figure 7A). Observed male germline enriched expression of young duplicated genes is interesting in the context of their acquisition and evolution. We perform the analogous RT-qPCR analysis of transcripts for *Esf2/ABP1* genes in selected tissues of *D. sechellia* adult males and females. In whole, we found a similar expression pattern to *D. melanogaster*: a ubiquitous expression of the older parental gene, but male-specific and testis-biased expression of more young duplicated copies (Figure S4).

As we have shown above (Figure 3A), *woodpecker* repeats have amplified in the *D. melanogaster* genome in the pericentromeric heterochromatin region, which is located within the expanded germline-specific *AT-chX* piRNA cluster, one of the specialized genomic regions providing long transcript precursors for producing a bulk of small piRNAs in gonads [40,49]. It is shown that the *AT-chX* is one of the major piRNA clusters both in the testes and ovaries of *D. melanogaster* [35,40]. However, *woodpecker* repeats are annotated as genes, containing one intron and possessing the highest nucleotide and amino acid homology among themselves (more than 99%) (Figure 1 block 2) that likely resulted from gene conversion homogenizing these gene duplicates or from very recent tandem duplication. Taking into account their testis-specific expression (Figure 7B), we asked whether the amplification of *woodpecker* genes within active piRNA cluster leads to potential piRNA production in the gonads.

To gain insights into peculiarities of *woodpecker* repeat expression, we analyzed profiles of small RNAs in the libraries from gonads of wild-type lines of *D. melanogaster*. Here we analyzed “locally unique” reads that map only to the region containing *AT-chX* and *woodpecker* repeats (see Materials and Methods for details). We found that multiple piRNA reads selected by size (23–29 nt) from the ovarian small RNA library were mapped to genome coordinates corresponding to the region. Surprisingly, piRNA reads mapped to *woodpecker*-containing region were not practically observed in the testis small RNA library (Figure 7C). Ovarian sense and antisense *woodpecker*-mapped piRNAs are distributed along the whole length of the *woodpecker* repeat consensus sequence with permitting 0–2 mismatches (Figure 7D), indicating that this genomic region is actually a part of the *AT-chX* piRNA cluster in the ovaries. Thus, *woodpecker* repeats function as a part of the double-stranded piRNA cluster in a sex-dependent manner only in the ovaries of *D. melanogaster*.

The repression of transposon activity is considered a main function of the piRNA pathway in *Drosophila* [41]; however, a cohort of piRNAs has been shown to take part in the repression of protein-coding genes [35,40,53]. Does the presence of antisense *woodpecker*-derived piRNAs indicate the possibility of silencing of *Esf2/ABP1* family genes in the ovaries of *D. melanogaster* due to high complementarity to their transcripts? To evaluate the potential feasibility of piRNA-mediated silencing, we independently mapped ovarian piRNA reads to transcript sequences of *woodpecker, cuckoo, brambling,* and *chaffinch* genes with the indicated number of mismatches, from 0 to 2 (Table 2). Evidently, the most read numbers were found to be mapped to *woodpecker* repeats themselves (201.6 rpm for sense, 568.6 rpm for antisense). The calculated ping-pong signature value (z_10_ score) suggested that the ping-pong amplification mechanism [54] is active in the biogenesis of these piRNAs in the ovaries. We also found that sense and antisense piRNA reads can be mapped to *brambling* and *cuckoo* transcripts (Table 2; Figure 7). Only a small number of piRNA reads was perfectly mapped (with 0–1 mismatch) to these transcripts, indicating piRNA origin from the distinct region. For transcripts of both *chaffinch* genes, we did not observe perfectly mapped piRNAs above background level (Table 2). Considering that target silencing requires a significant number of antisense piRNAs with a high level of complementarity to the target [35,40], we assumed that these piRNAs could presumably repress expression of *woodpecker* and *brambling* genes in the ovaries. However, piRNA silencing potential for the repression of *cuckoo,* which is the only *Esf2/ABP1* paralog expressed in the ovaries, appears to be not enough, because the level of mapped antisense piRNAs is less than 100 rpm (Table 2). Our comparative analysis of polyA + RNAseq libraries of gonads of *aub* mutants (with disrupted piRNA biogenesis) and their heterozygous siblings revealed no significant change in the expression of all *Esf2/ABP1* paralogs in the gonads, suggesting that they are not regulated by piRNAs (Figure 7E). In the ovaries of *aub* mutants, the total amount of piRNAs drops more than 35-fold, while *woodpecker*-mapped piRNAs are not produced (Figure 7E).

## 4. Discussion

Here we provide analysis of the evolutionary history of *Esf2/ABP1* gene duplications in *Drosophila* species of the subgenus *Sophophora.* Using high-quality genome assemblies and synteny analysis, we identified 56 homologous *Esf2/ABP1* genes in 16 species of the *melanogaster* group, separating approximately 25 MYA of evolution (Figure 1 and Figure 2, and Figure S1). Our analysis revealed that despite the single copy of the *Esf2/ABP1* gene per genome harbored in the majority of *Drosophila* species, gene duplication events occurred in four species of the *melanogaster* subgroup and independently in three species of the *suzukii* subgroup (Figure 2 and Figure S1, Table S1). We found that recurrent DNA-mediated duplication events occurred at the same cytolocation nearby the parental copy as well as with translocation and amplification of duplicated gene copies in the introns of unrelated genes or in heterochromatic piRNA cluster (Figure 2).

**Figure 8:**
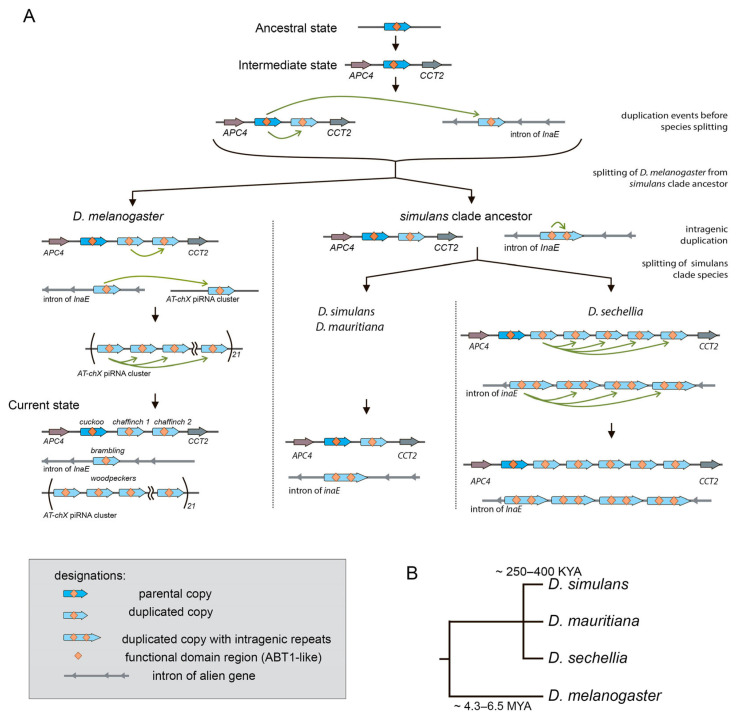
(**A**) Reconstruction of the basic events of the emergence of duplicated copies of *Esf2/ABP1* genes in the *melanogaster* subgroup genomes based on phylogenetic and functional domain analysis. A single *Esf2/ABP1* (parental) copy is located on the X chromosome flanked by *APC4* and *CCT2* genes in the genome of an intermediate ancestor fly. Duplication events indicated here by green arrows occurred both before and after speciation. Duplicated copies of the *simulans* clade species contain intragenic duplication, encoding ABT1-like domain. (**B**) The cladogram exhibits the relationship between *D. melanogaster* and the *simulans* clade species. *D. melanogaster* splits from the common ancestor with the *simulans* clade between 4.3 and 6.5 million years ago. *D. simulans*, *D. mauritiana*, and *D. sechellia* diverged from each other near 250–400 thousand years ago.

Our phylogenetic and functional domain analyses (Figure 4 and Figure 5) allow us to reconstruct the evolutionary history of *Esf2/ABP1* gene duplications in *D. melanogaster* and its sibling species of the *simulans* clade (Figure 8A). According to the proposed scenario, duplication events took place before the speciation of *D. simulans* and *D. mauritiana*, whereas they occurred both before and after speciation in *D. melanogaster* and *D. sechellia*, providing the emergence and fixation of multiple new genes encoding proteins with RNA-binding ABT1-like domain (Figure 5). Duplicated paralogs in *D. melanogaster* do not exhibit any traces of pseudogenization and possess the characteristic features of functional copies. They contain an unbroken ORF, one conserved intron sequence, and an RNA-binding domain (ABT1-like) encoding region (Figure 5 and Figure S2). We found that they acquired predominantly male-specific and testis-enriched expression pattern, unlike the parental gene, *cuckoo* (*CG32708*), which is expressed in different male and female tissues, with the highest expression in the ovaries and testes (Figure 7A,B). Among them, two duplicated copies in the same location with the parental gene, *chaffinch 1* (*CG32706*) and *chaffinch 2* (*CG6999*), have diverged considerably from *cuckoo*; however, they were found to maintain a high identity to each other (Figure 1 block 4 and Figure 4).

The fate of most gene duplications is a rapid deletion or pseudogenization, but in some cases a duplicate copy can be fixed in the genome by drift or selection during a so-called fixation phase and subsequently maintained over time owing to acquiring a benefit function in the organism [6]. More than a dozen models were developed to explain the emergence, evolution, and maintenance of gene duplications in genomes [2,5,6]. Note that the parental gene *cuckoo* is essential for fly development and cell proliferation in *D. melanogaster* [9,10,11,12]. *cuckoo* has a high nucleotide and protein identity with orthologous genes of other species of the *melanogaster* group in the syntenic location (Figure 1 block 1 and Figure 2, Table S1) and is maintained under purifying selection in the genome (Table S3). Molecular functions of *chaffinch 1* and *chaffinch 2* genes are not understood to date; however, we determined the distinct pattern of *chaffinch* gene expression, i.e., male-biased expression with a maximal level in the testes, and the absence or very low expression level in female tissues (Figure 7A,B). It allows excluding subfunctionalization or duplication–degeneration–complementation models from consideration, because in these cases expression pattern must be shared between the subfunctionalized original and duplicated copies. According to the neofunctionalization model, duplicated copies occasionally accumulate substitutions to acquire a novel gene function that will be supported by selection, whereas a parental copy will not. Due to the conservation of the whole exon–intron structure and functional domain of *chaffinch* genes (Figure 5), we do not consider the neofunctionalization model as permissible. The Z-test for positive selection for both *chaffinch* genes showed no deviation from neutrality (Table S3). We proposed that the most suitable model for the evolution of *chaffinch* genes is the “beneficial increase in dosage”. As the heterogametic sex in *Drosophila*, males possess a gene-rich X chromosome and a strongly heterochromatic Y chromosome with few protein-coding genes [55]. To correct the potentially lethal imbalance of X-linked gene expression level in males, the mechanism of dosage compensation in somatic cells of *D. melanogaster* males provides an approximately two-fold increasing expression of most X-linked genes with the aid of attraction of the MSL (Male-Specific Lethal) protein complex to the male X chromosome for acetylation of histone H4 at K16 position (acetylH4K16), a mark of active chromatin [56,57]. However, the somatic dosage compensation system appears to be not applicable to male germline cells, where MSL complex is not assembled and the chromatin of the X chromosome loses a high level of acetylH4K16 modification [58,59]. It is shown that testis germ cells in *Drosophila* are characterized by a massive wave of transcription on the spermatogonial stage but undergo meiotic X chromosome inactivation later on the stage of mature spermatocytes [60]. Taking all these circumstances into account, we propose that owing to the extra transcript level in the period of active transcription in the testes, these X-linked gene duplications have been fixed and retained by a dosage selection, according to the beneficial increase in gene dosage model.

The same logic appears applicable to the remaining duplicated *Esf2/ABP1* genes in *D. melanogaster*, *brambling*, and *woodpeckers*, since they all support a strong testis-restricted expression, preserve the exon–intron structure, and retain a functional ABT1-like domain (Figure 5 and Figure 7A,B). *woodpecker* tandem gene amplification is unique to the *D. melanogaster* genome. According to our phylogenetic analysis, *brambling* and all *woodpecker* genes form the monophyletic group on the tree (Figure 4), confirming the origin of the last ones from the single-copy *brambling* gene after speciation of *D. melanogaster* (Figure 8A). The preservation of nearby 1 kb promoter region of *woodpecker* genes with 95% sequence homology with the *brambling* promoter serves as the additional confirmation for our proposition. We could assume that both *brambling* and *woodpecker* genes were fixed and preserved in the genome also owing to the beneficial increase in gene dosage. However, in the case of *woodpecker* genes, their genomic location leads to unexpected functional consequences that likely require a correction of the proposed model. The introduction of young duplicated copies into the piRNA cluster (Figure 2 and Figure 3A) resulted in their ovary-specific functional innovation. While in the testes these repeats are expressed as protein-coding genes, providing increased gene dosage; in the ovaries these repeats are subject to double-stranded non-canonical transcription as long precursors for the piRNA biogenesis (Figure 7C,D). Determination of the forces ensuring the preservation of highly identical *woodpecker* genes in this unusual genomic location appears to be complex. To date we did not find evidence of transposon involvement in the initial insertion of *woodpecker* into the piRNA cluster. However, we assume that the insertion of the *woodpecker* copy in the region enriched by transposon fragments (Figure 3A) could facilitate the following amplification of *woodpecker* genes owing to unequal homologous recombination. Repetitive transposon fragments flanking the insertion may represent primary sites that mediated homologous recombination. At the same time, the mechanism of gene conversion can impact *woodpecker* maintenance in the highly homologous state.

It is known that piRNA-mediated target repression requires near-perfect complementarity of antisense piRNAs [35,40,61] to prevent off-target silencing. We found that piRNA silencing potential of *woodpecker*-derived antisense piRNAs for repression of *cuckoo,* which is the only *Esf2/ABP1* paralog with detected expression in the ovaries, is rather weak owing to small amount of piRNAs with perfect complementarity to *cuckoo* transcripts (Table 2). However, we cannot exclude that this piRNA amount may be sufficient to down-regulate the expression of *cuckoo* at a certain stage of oogenesis in a limited number of cells. The capacity of these piRNAs to target protein-coding genes warrants future investigations. It has been shown that the piRNA program in *D. melanogaster* gonads is sexually dimorphic [35,62]. However, upstream mechanisms providing this sexual dimorphism are weakly studied. To date, it is still difficult to determine the exact forces that drive piRNA cluster formation and evolution and what circumstances transform a part of the active piRNA cluster in a protein-coding genomic locus in the sex-dependent manner. These circumstances can include the regulatory role of the three-dimensional level of chromatin organization, the specific modifications of histones across the locus, including me3H3K9, the attraction of sex-specific chromatin complexes and factors, and the presence of flanking insulator sequences. In summary, here we uncovered a case of the functional adaptation of duplicated non-transposon genes with their sex-specific transformation into non-coding small RNAs. The acquisition of this potentially sex-antagonistic novel function took place owing to the insertion and amplification in a new genomic context without a significant divergence of *woodpecker* repeats from their parental *brambling* gene.

Note that a related history has been reconstructed earlier for the origin, evolution, and maintenance of the *Stellate-Suppressor of Stellate* (*Ste-Su(Ste)*) genetic system in the *D. melanogaster* genome [63,64]. Tandem amplification arrays of highly homologous genes, *Stellate* and *Su(Ste)*, arose from a chimeric Y-chromosomal intermediate precursor on the X and Y chromosomes, respectively. The emergence of *Su(Ste)* repeats in heterochromatin context as well as the insertion of transposon *hoppel* in the *Su(Ste)* promoter appear to facilitate *Su(Ste*) repeat pseudogenization and the acquisition of bidirectional transcription across them, providing abundant *Su(Ste)* piRNAs for the silencing of harmful *Stellate* genes. *Su(Ste)* repeats have lost protein-coding potential but evolved to form the major testis-specific piRNA cluster in *D. melanogaster* genome [40,53], ensuring *Stellate* gene repression and maintenance of correct spermatogenesis. Thus, *woodpecker* repeats, which are proposedly at an earlier stage of their evolution than the *Ste-Su(Ste)* system, can be considered as a valuable model for studying the evolutionary forces driving the formation of piRNA clusters.

In relation to the pattern of duplications in species of the *simulans* clade, we can note that according to our hypothesis (Figure 8A), in *D. simulans* and *D. mauritiana* genomes the parental gene and both duplicated copies were inherited from an immediate ancestor of the clade. In *D. sechellia*, additional amplification events supposedly happened after speciation. Considering a short divergence time between the *simulans* clade species (Figure 8B), we can suggest that amplified copies of *D. sechellia* located in cluster 1 and cluster 2 are very young. They maintain extremely high identity between each other within their genome locations (Figure 1 blocks 3 and 4). Analyzing the expression pattern of *Esf2/ABP1* genes in the selected tissues of *D. sechellia* adult males and females, we revealed a picture strikingly similar to *D. melanogaster*, with testis-enriched expression of young duplicated copies and a ubiquitous expression of the parental gene (Figure S4). Considering intragenic duplication with the acquisition of the second RNA-binding ABT1-like domain by the duplicated copies of cluster 2 (Figure 5), we can propose that testis-specific dosage selection also took place for their retention and maintenance.

We uncovered several duplication events for *Esf2/ABP1* genes in the *suzukii* subgroup species (Figure 2). Despite that in both the *melanogaster* and *suzukii* subgroup species we observed distribution of *Esf2/ABP1* family genes only on the X chromosome, the duplication events in these subgroups likely occurred independently through different mechanisms (Figure 2, Table S1). A search of functional domains for Esf2/ABP1 proteins of the *suzukii* subgroup allows us to reveal an unexpected domain diversification. These Esf2/ABP1 proteins, in addition to ABT1-like domain preservation, contain long Intrinsically Disordered Regions, IDRs, composing 50 to 80% of the whole polypeptide length (Figure 6, Figures S2 and S3). They are significantly longer than those of *melanogaster* subgroup orthologs (Figure 5 and Figure 6). IDRs are generally described as protein regions that do not adopt a defined three-dimensional structure under physiological conditions, but they are nevertheless functional. It is known that the majority of eukaryotic proteins contain both structured and disordered regions; however, only about 10% of proteins consist of IDRs representing half or more of their length, relating to the category of highly disordered proteins [52]. Among the Esf2/ABP1 family proteins of the *suzukii* subgroup species, we determined two, LOC119556661 of *D. subpulchrella* and LOC108023671 of *D. biarmipes*, containing multiple intragenic tandem repeat sequences that contribute to a great extension of their continuous IDRs to near 80% of their whole length, consisting of 534 aa and 759 aa, respectively (Figure 6 and Figure S3). Note that these tandem repeats are strongly enriched by acidic amino acid and serine residues, in sum consisting of more than 40% of their content (Figure S3). A common feature of long IDRs is their ability to form dynamic multivalent, flexible, and self-aggregating molecular networks, providing the assembly of liquid–liquid phase-separated droplets or hydrogels, commonly named biomolecular condensates [52,65]. It is shown that expanded IDRs of certain RNA-binding proteins enable the formation in the cells of macromolecular granule-like assemblies, allowing localization, accumulation, and storage of RNAs [52,65,66]. Although the verification of molecular functions of expanded IDRs in fly tissues needs further investigation, we propose that Esf2/ABP1 proteins in the *suzukii* subgroup species evolved with the acquisition of novel functional features mediated by their IDRs for RNA accumulation and processing. The tandem expansion of internal repeats is one of the ways by which IDR-encoding genes arose and spread during evolution [67]. According to our findings, we assume that intragenic duplications and fragment shuffling by recombination were presumable selection instruments driving the evolution of *Esf2/ABP1* family genes in a common ancestor of the *suzukii* subgroup flies. After species divergence, the expansion of IDRs owing to multiple internal repeats independently in *Esf2/ABP1* copies of *D. subpulchrella* and *D. biarmipes* appears to contribute to following evolutionary adaptation. However, the evolutionary scenario for this subgroup can hardly be accurately reconstructed owing to the complicated pattern of arising and loss of *Esf2/ABP1* genes (Figure 2).

## 5. Conclusions

To investigate the evolutionary history of *Esf2/ABP1* family duplicates in *Drosophila*, we employed a multifaceted approach, combining comparative genomics, phylogenetic analysis, functional domain analysis, and tissue-specific transcriptomic analysis. This strategy allowed us to identify and characterize 47 duplicated gene copies across seven *Drosophila* species clustered at three distinct genomic locations on the X chromosome. Recurrent gene duplication events occurred in four species of the *melanogaster* subgroup and independently in three species of the *suzukii* subgroup. In both cases duplication provided the emergence and fixation of multiple new genes encoding proteins with RNA-binding ABT1-like domain. In three species of the *suzukii* subgroup, *Esf2/ABP1* genes evolved with domain diversification: in addition to RNA-binding ABT1-like domain preservation, all homologous proteins acquired expanded Intrinsically Disordered Regions.

The first and the second duplication events in four species of the *melanogaster* subgroup probably occurred before the splitting of *D. melanogaster* from the species of the *simulans* clade, but the third one occurred after. Gene, named *cuckoo*, and its orthologs are parental, characterized by ubiquitous expression in different fly tissues. The *chaffinch 1* and *chaffinch 2* genes, resulting from a second duplication event, have a distinct pattern of expression, i.e., male-biased expression with a maximal level in the testes. We proposed that the most suitable model for the evolution of *chaffinch* genes is the “beneficial increase in dosage”. Purifying selection maintains the parental copy, *cuckoo*. In relation to other duplicated copies in all three genomic locations, we did not find significant deviations from neutral evolution. The insertion and amplification of young duplicated *woodpecker* copies into the piRNA cluster lead to sex-specific functional innovation. Whereas in the testes these repeats are expressed as protein-coding genes; in the ovaries they undergo non-canonical bidirectional transcription as long piRNA precursors.

This way, used complex approach permitted us to reconstruct duplicated gene evolutionary trajectories and revealing how their functions and expression patterns diverged over time.

## Figures and Tables

**Table 1:** The list of new designations for *Esf2/ABP1* family genes in *D. melanogaster*.

Gene Number	New Gene Designation
*CG32708*	*cuckoo*
*CG32706*	*chaffinch 1*
*CG6999*	*chaffinch 2*
*CG10993*	*brambling*
*CG40813*	*woodpecker 1*
*CG46504*	*woodpecker 2*
*CG46505*	*woodpecker 3*
*CG46506*	*woodpecker 4*
*CG46507*	*woodpecker 5*
*CG46508*	*woodpecker 6*
*CG46509*	*woodpecker 7*
*CG46510*	*woodpecker 8*
*CG46511*	*woodpecker 9*
*CG46512*	*woodpecker 10*
*CG46513*	*woodpecker 11*
*CG46514*	*woodpecker 12*
*CG46515*	*woodpecker 13*
*CG46516*	*woodpecker 14*
*CG46517*	*woodpecker 15*
*CG46518*	*woodpecker 16*
*CG46519*	*woodpecker 17*
*CG46520*	*woodpecker 18*
*CG46521*	*woodpecker 19*
*CG46522*	*woodpecker 20*
*CG41562*	*woodpecker 21*

**Table 2:** Result of independent mapping of piRNA reads from *Oregon R* ovarian library to *woodpecker*, *cuckoo*, *brambling,* and *chaffinch* genes with the indicated mismatch number. The number of 10 nt overlapping of sense and antisense piRNA pairs and the z_10_ score are indicated in the two bottom lines, respectively. NA—data not available.

Gene	*woodpeckers*		*brambling*		*cuckoo*		*chaffinch1*		*chaffinch2*	
piRNA Read Type	Sense piRNAs	Antisense piRNAs	Sense piRNAs	Antisense piRNAs	Sense piRNAs	Antisense piRNAs	Sense piRNAs	Antisense piRNAs	Sense piRNAs	Antisense piRNAs
Mapped reads (0–2 mm), rpm	201.6	568.6	115.8	203.8	68.8	47.2	0.6	0.2	0	0.2
Mapped reads (0 mm), rpm	150.3	422.7	1.1	55.2	15.9	0.9	0	0	0	0
Mapped reads (1 mm), rpm	40.5	129.5	25.2	51.4	10.1	3.2	0	0	0	0
Mapped reads (2 mm), rpm	10.7	16.4	89.5	97.1	45.7	43.2	0.6	0.2	0	0.2
Overlap_10, pairs	365		155		23		0		0	
z_10_ score	2.86		3.08		1.01		NA		NA	

## Data Availability

The open-source RNAseq data resources list used in this study is included in the Supplementary Materials, Table S2.

## Supplementary Materials

The following supporting information can be downloaded at https://www.mdpi.com/article/10.3390/insects16090956/s1; Figure S1: Phylogenetic tree of Esf2/ABP1 family orthologs and paralogs in the genus Drosophila; Figure S2: Alignment of RRM ABT1-like domains predicted in Esf2/ABP1 family proteins of the melanogaster subgroup and suzukii subgroup; Figure S3: Representation of multiple tandem intragenic repeats for proteins LOC119556661 of *D. subpulchrella* (6.5 repeats) (A) and LOC108023671 of *D. biarmipes* (11 repeats) (B); Figure S4: RT-qPCR analysis of transcript levels of Esf2/ABP1 family genes in the gonads, carcasses, and heads of adult male and female flies of *D. sechellia*; Table S1: The table contains data for determination of orthologs and paralogs of Esf2/ABP1 gene family in genome assemblies of Drosophila species; Table S2: List of RNA library resources used for the analyses; Table S3: A codon-based test of neutrality, the Z-test, for selection type for the *Esf2/ABP1* genes of *D. melanogaster* was performed using MEGA software.
