# Supplementary material for: Recurrent Duplication, Testis-Biased Expression, and Functional Diversification of Esf2/ABT1 Family Genes in Drosophila
Source: Insects. 2025 Sep 11;16(9):956. doi: 10.3390/insects16090956 (PMC12470439; doi:10.3390/insects16090956)
Supplement: Supplementary file 1 [file insects-16-00956-s001.zip › Supplementary Figures S1-S4 Table S3.pdf]

Supplementary information of research article

Recurrent Duplication, Testis-Biased Expression, and Functional Diversification of *Esf2/ABT1* Family Genes in *Drosophila*

Elizaveta D. Davydova [1,†], Alexei A. Kotov [1,†], Alina V. Chernizova [2], Ekaterina Yu. Yakovleva [1] and Ludmila V. Olenina [1,*]

[1] Laboratory of Functional Genomics, Koltzov Institute of Developmental Biology, Russian Academy of
Sciences, 26 Vavilov Str., 119334 Moscow, Russia; elizaveta.dav@yandex.ru (E.D.D.), kotov_alexei@mail.ru (A.A.K.); e.u.yakovleva@gmail.com (E.Y.Y.)

[2] Laboratory of Cellular Neurobiology of Learning, Institute of Higher Nervous Activity and Neurophysiology, Russian Academy of Sciences, 5A Butlerov Str., 117485 Moscow, Russia; alisach64@gmail.com

* Correspondence: olenina_ludmila@mail.ru

† These authors contributed equally to this work.

The file contains Figures S1-S4 with figure legends and Table S3.

Figure S1

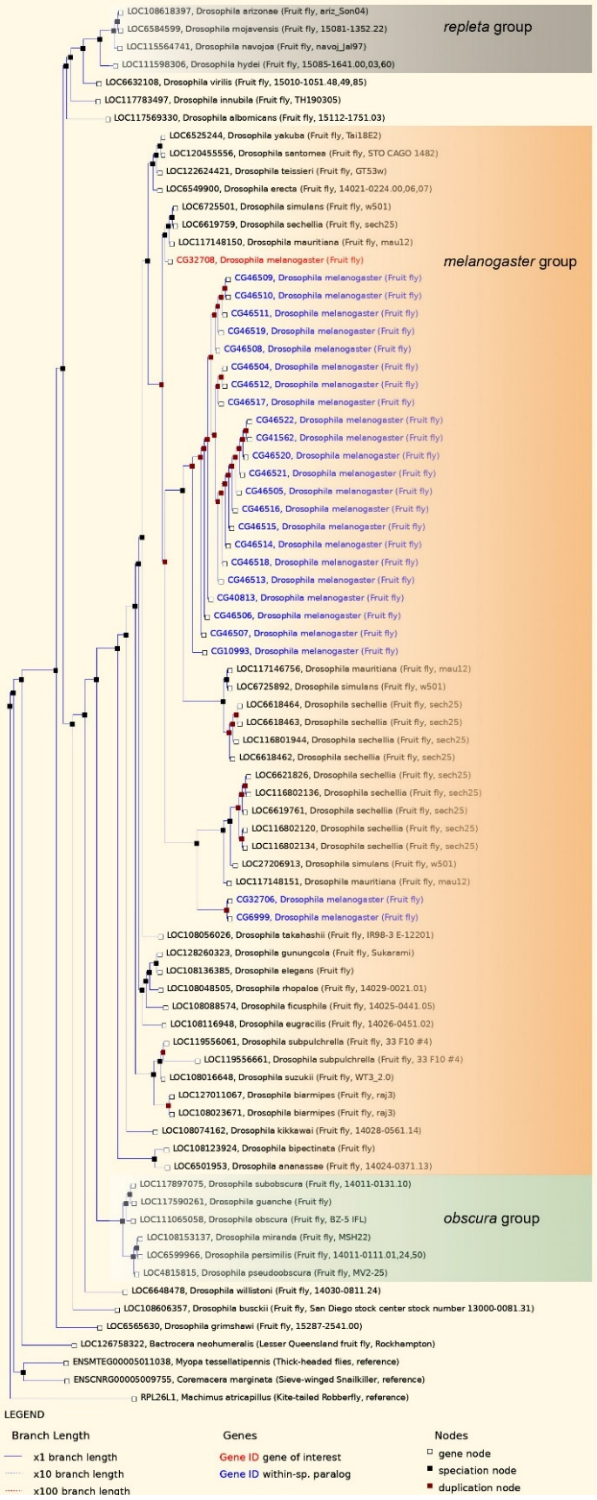

Figure S1. Phylogenetic tree of *Esf2/ABP1* family genes in the genus *Drosophila*. Only a single copy per genome of *Esf2/ABP1* genes is found across the majority of *Drosophila* species. Duplication events were designed by red square nodes. The tree was built using Ensemble Metazoa database and tools (https://metazoa.ensembl.org/). Homologs from *Coremacera marginata, Myopa tessellatipennis,* and *Bastrocera neohumeralis* Diptera flies were used as outgroups for the tree generation. Gene *RPL26L1* from *Machimus atricapillus* was used as an unrelated sequence. Genome assembly designations are indicated for all species except *D. melanogaster* (Release 6 plus ISO1 MT assembly). Note that duplications in the *D. suzukii* genome were not determined by this analysis and were observed later (see main text).

Figure S2

protein	domain start position	sequence	domain finish position
Consensus seq ABT1	01	GIVYLSRIPPGMNPAKLRQLLS-YGEVGRIYLQPE------------------FTEGWVEFEDKKVAKRVAESLN--PIGGKKRSRFRDDLWNIKYLP	98
D_mel CG32708	74	GIIYISNIPKHMNVTRLREILGEYGAIGRVYLQPEKLSSAKAKKNKRKRYNIHFTEGWVEFKSKRVAKQIVPLLNNKQISGRKTSQFYDSLWSMKYLP	171
D_mel CG32706	61	GVILISNIPKDMDGDCLKEIMNLHSVVGRVYVQPETLSSFKTKKNMRK--------GWVEFISKSGAKKIALELNNKPITDGKSSRFRGLLWKMKFLP	150
D_mel CG6999	61	GVILISNIPKDMDGDCLKEIMNLHSVVGRVYVQPETLSSFKTKKNMRK--------GWVEFISKSGAKKIALELNNKPITDGKSSRFRGLLWKMKFLP	150
D_mel CG10993	65	GVIYISNLPKHMTLTRLREILGEYGAIGRAFLRSQKLS--------RKPHNFLFAEGWVEFKSKRVAKQIVPLLNYKQISTHKKSPFYYSLWIMEYLP	164
D_mel CG46513	75	GIIYISNIPKHMTLTRLREILGEYGGIGRAFLRSQSSKHH----------NILFAEGWVEFKSKHVAKQLVLLLNNKQISTRNNSPFYYSLWSMEYLP	157
D_sim LOC6725501	74	GIIYISNIPKHMNVTRLREILGEYGTIGRVYLQPEKLSSAKAKKNKRKRYNIHFTEGWVEFKSKRVAKQIVPLLNNKQISGRKTSQFYDSLWSMKYLP	171
D_sim LOC27206913	56	GVITISNVPKDMSDMRLYKIMQMHSLIGRVYVQPKELSSFKTNNNQRK--------GWVEFISKSGAKKIARELNNKPITRNISSPFYGLLWNMKFLP	145
D_sim LOC6725892	71	GTIYISHLPKHMALTSVLQIFGEYGAIGRAFLRSKKLSSKSPDIL--------FTEAWVQFNSKCVAKQIVPLLNNKQISTHKNSKFYDSLWSMKYLP	160
D_sim LOC6725892	239	GIIYICCIPKDMTMARLREILGKYGAVGRAYLQSQKLSDLSPHII--------FAEGWVEFKSKRVARQIVPLLNNRQISTHKKSRFYDSLWSMKYLP	328
D_mau LOC117148159	74	GIIYISNIPKHMNVTRLREILGEYGTIGRVYLQPEKLSSAKAKKNKRKRYNIHFTEGWVEFKSKRVAKQIVPLLNNKQISGRKTSQFYDSLWSMKYLP	171
D_mau LOC117148151	48	GVITISNVPKDMTDLRLYEIMRMHSAIGCVYVQPKELSSFKTNNNQRK--------GWVEFISKSGAKKIARELNNKPITGNKSSPFYGLLWNMKFLP	137
D_mau LOC117146756	110	GTIYISHLPKHMAVTNMLRIFGEYGAIGRAFLRSKKLSSKSPD--------ILFTEGWVQFNSKRVAKQIVPLLNNKQISTHKKSRFYDSLWSMKYLP	199
D_mau LOC117146756	278	GIIYICNIPKNMTMARLREILGQYGAIGRAYLQSQKLSDKSPH--------IIFAECWVEFKSKRLAKQLVILLNNRQISPHKKSRFYDSLWHMKYLS	367
D_sech LOC6619759	74	GIIYISNIPKHMNVTRLREILGEYGTIGRVYLQPEKLSSAKAKKNKRKRYNIHFTEGWVEFKSKRVAKQIVPLLNNKQISGRKTSQFYDSLWSMKYLP	171
D_sech LOC6621826	55	GVITISNVPKDMTVLRLYEIMGMHSLIGRVYVQPKELLSFKTNNNHRK--------GWVEFISKSGAKKIARELNNKPISRKTSSPFYGLLWNMKFLP	144
D_sech LOC6618463	69	GTIYISHLPKDMAVTSMLRIFGEYGAIGRAFLRSKKLSS--------KSPDIIFTEGWVQFNSKRVAKQIVPLLNNKQISTRKKSRFYDSLWSMKYLP	158
D_sech LOC6618463	212	GIIYICNIPKDMTIARLREILGKYGAVGRAYLQSQKLSD--------KSPHIIFAEGWVEFKSKRVAKQIVPLLTNRQISTHKKSRFYDSLWSMKYLS	301
D_yak LOC6525244	69	GIIYISNIPKHMNVTRLREILGEFGTIGRVYLQPEKLSSAKAKKNKRKRYNIHFTEGWVEFESKRVAKQIVPLLNNKQISTRKKSQFYDSLWSMKYLP	166
D_suz LOC108016648	127	GIIYISNIPKHMNVTRLREILGEYGKIGRVYLQPEKLSSAKAKKNKRKRYNIHFTEGWVEFESKGIAKYLVTVLNNSKISTRKKSQFYDSLWSMKYLP	224
D_suz LOC108016563	89	GIIYMSNVPKHLKVKRMRKILKKFGKIGKIILQP----SAKAKRNKRKR--IHFYEGWVEFESNEIAKFVATALGNSRISKHKSSRFYDSLWRMKYLH	180
D_sub LOC119556661	122	GIIYISNIPKHMNVTRLREILGEYGKIGRVYLQPEKLSSAKAKKNKRKRYNIHFTEGWVEFESKGIAKYLVTVLNNSKISTRKKSQFYDSLWSMKYLP	219
D_sub LOC119556061	542	GIIYISNIPKHMNVTRLRKILGEYGKIGRVYLQPEKLSSAKAKKNKRKGRKIRFTEGWVEFESKGIAQFLVTILNNSKISNRKKSKFSDSLWSMKYLS	639
D_bia LOC127011067	162	GIIYISNIPKHMNVTRLREILGEYGKIGRVYLQPEKLSSAKAKKNKRKRYNIHFTEGWVEFESKGIAKFLATVLNNSKISTRKKSQFYDSLWSMKYLP	259
D_bia LOC108023671	848	GIIYISNIPKHMNVTRLREILGEYGKIGRVYLQPEKLSSAKAKKNKRKRYNIHFTEGWVEFESKGIAKFLATVLNNSKISTRKKSQFYDSLWSMKYLP	945

Figure S2. Alignment of RRM ABT1-like domains predicted in Esf2/ABP1 family orthologs and paralogs of the *melanogaster* subgroup and the *suzukii* subgroup. For domain determination, a search in the NCBI's Conserved Domain Database (CDD) (https://www.ncbi.nlm.nih.gov/Structure/cdd/wrpsb.cgi) was used. The search was performed against database CDD v3.21 with an expected value threshold of 0.01. The consensus sequence of the RRM ABT1 domain presented on the top was built for proteins from multiple species by CDD tools. Note that only one protein sequence of CG46513 (Woodpecker 11) is present for 21 Woodpecker proteins of *D. melanogaster*, LOC6621826 is present for practically identical duplicated proteins of *D. sechellia* (LOC6621826, LOC116802136, LOC116802134, LOC116802120, LOC6619761) of cluster 1, and LOC6618463 is present for proteins of cluster 2 (LOC6618463, LOC116801944, LOC6618464, LOC6618462).

Figure S3

A

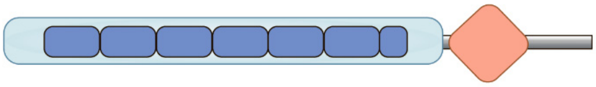

LOC119556661 translated CDS D. subpulchrella

MPKAEKNKKQKQDPAPI

EQLFSSEDDYDMNLANFQRGSKSAPTKPKPEEQHASSDDENNFNLVNIIKGTKPQKASLSESESESGEEPSDESELEPVEQDA

EQFLFSDDGYDMNLANFERGSKSAPTKPKPEEQDTSSDDENKLYLP------KPQKASLSESESEEEPSDESDSELK--EQDA

EQFLFSDDGYDMNLANFERGSKSAPTKPKPEEQDTSSDDENKLYLP------KPQKASLSESESEEEPSDESDLE--PVEQDA

EQFLFSDDGYDMNLANFERGSKSAPTKPKPEKQDTSSDDENKLYLP------KPQKASLSESESEEEPSDESDSELK--EQDA

EQFLFSNDGYDMNLANFKRGSKSAPTKPKPEEQDASSDDENKLYLP------KPQKASLSESESEEEPSDESDSELK--EQDA

EQFLFSDDGYDMNLANFKRGSKSAPTKPKPEKQDTSSDDENKLYLP------KPQKASLSESESEEEPSDESDSELE--EQDA

EQPLSSDGDYDMNLANFKNGTKSVPTKPEP-KKKY--D---KLPVP

tandem intragenic repeats

SDDEDEMDMANFKNGSTPARTKKAKK**GIIYISNIPKHMNVTRLRKILGEYGKIGRVYLQPEKLSSAKAKKNKRKGRKIRFTEGWV**

EFESKGIAQFLVTILNNSKISNRKKSKFSDSLWSMKYLSCFKWVHLTERLNYEQNNLIKNEFLKKLAKKPKAEKFGNAAKKMKMT

TTQSE

B

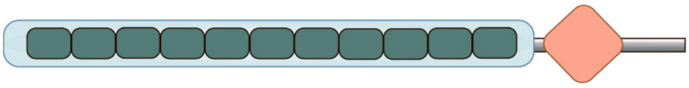

LOC108023671 translated CDS Drosophila biarmipes

MLKVKKNKKPKQQPAPIEMEESESEEASLSESEPEKEPSDESETPEEEPSSDSEPEQEPSDESDIEPEEQDASSDDDEIIKLGNG

RNGTGPEEATSGESEPENELAYESDSEPEEQDASSDDDKTDLGKFNNVKKPWKGYLSESEAETENSDESDSDNDSEILYLNFKEE

QVAEASSSQSESE

KASLNESEPEKEPSDESDLEPEEHGTSSADTDDKNLATSEDYKMGLAE

NGKKPKKAVLSESEPENEPSDESDLEPEERGASSDDADYKNLATSEDYKMGLAE

NGKKPKKAVFSESEPENESSDESDLEPEERGASSDDADYKNLALSKDNKMDLAK

NGKKPKKAVLSESEPENEPSDESDLEPEERGASLDDADYKNLALSKDNKMGLAE

NGKKPKKAVWSESEPEDEPSDESDLEPEERGASSDDADYKNLALSKDNKMGLAE

NGKKPKKAVWSESEPENEPSDESDLEPEERGASSDDADYKNLASSKDNKMGLAE

NGKKPKKAVWSESEPENEPSDESDLEPEERGASSDDADYKNLASSKDNKMGLAE

NGKKPKKAVLSESEPENEPSDESDLEPEERGASSDDADYKNLALSKDNKMGLAE

NGKKPKKAVLSESEPENEPSDESDLEPEERGASSDDADYKNLALSKDIKMDLAK

NRKKPKKAVFSESEPENESSDESDLEPEERGASSDDADYKNLALSKDNKMDLAK

NGKKPKKAVFSESEPENEPSDESDLEPEERGASSDDADDTNIANFK

tandem intragenic repeats

NGSKSDRTKPKPKEQDAELPISSEDDDEMDLANFKNGSRSTRVKPAKKAKR**GIIYISNIPKHMNVTRLREILGEYGKIGRVYLQP**

EKLSSAKAKKNKRKRYNIHFTEGWVEFESKGIAKFLATVLNNSKISTRKKSQFYDSLWSMKYLPRFKWVHLTERMNYEQAVHKQR

LHTEVSQARKETTFFQNNLDKSDYLKKQAKKADKAETYEMPPKKKVKKAVSGSED

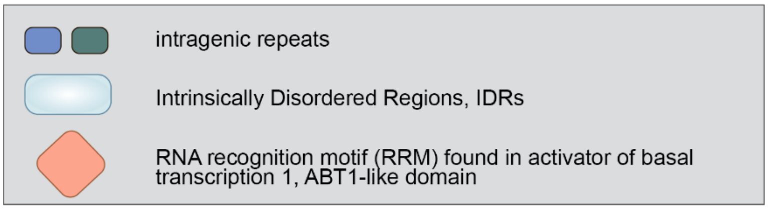

Figure S3. Representation of multiple tandem intragenic repeats for proteins LOC119556661 of *D. subpulchrella* (6.5 repeats) (**A**) and LOC108023671 of *D. biarmipes* (11 repeats) (**B**). The repeat sequences of the protein are aligned with each other. ABT1-like domains are marked by red letters in both sequences.

Figure S4

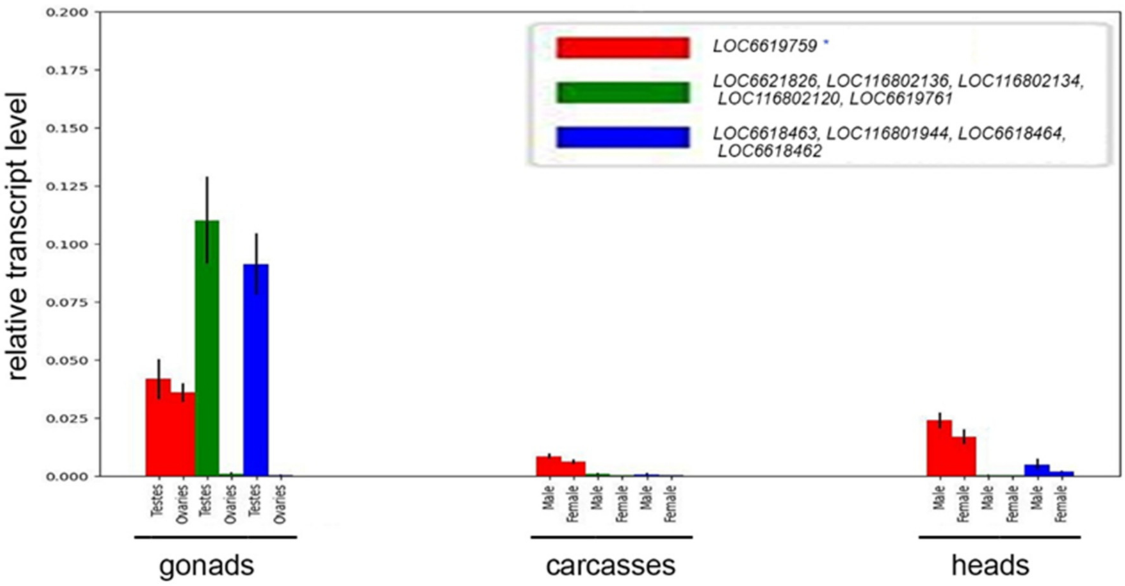

Figure S4. RT-qPCR analysis of transcript levels of *Esf2/ABP1* family genes in the gonads, carcasses, and heads of adult male and female flies of *D. sechellia*. A blue asterisk marks the parental gene copy. The aggregated expression levels present for highly identical duplicated copies from cluster 1 (*LOC6621826, LOC116802136, LOC116802134, LOC116802120, LOC6619761*) and cluster 2 (*LOC6618463, LOC116801944, LOC6618464, LOC6618462*). The expression levels were normalized to *rp49* transcripts. Error bars represent standard errors of the mean.

Table S3. A codon-based test of neutrality, the Z-test, for selection type for the *Esf2/ABP1* genes of *D. melanogaster was* performed using MEGA software. The test statistic (*dN - dS*) is represented as Z-score in the column, where *dS* and *dN* are the numbers of synonymous and nonsynonymous substitutions per site, respectively. Values of *P* less than 0.05 are considered significant and are marked by asterisks. We generally used orthologous genes of *D. simulans* or *D. yakuba* as outgroups for pairwise alignment generation. In certain cases, we used comparisons between *D. melanogaster* paralogs.

**analyzed gene of *D. melanogaster***	**gene for comparison**	**Z-score**	**P-value**	**type of selection**
cuckoo (CG32708)	*LOC6725501* (*D. simulans*)	3.44	4.1E-04*	purifying selection
	LOC6525244 (*D. yakuba*)	6.53	8.0E-10*	purifying selection
chaffinch 1 (CG32706)	*LOC27206913 (D. simulans)*	-0.764	0.446	neutral evolution
	chaffinch 2 (D. melanogaster)	0.708	0.489	neutral evolution
	cuckoo (D. melanogaster)	-0.798	0.426	neutral evolution
chaffinch 2 (CG6999)	*LOC27206913 (D. simulans)*	-0.931	0.354	neutral evolution
brambling (CG10993)	*LOC6725892 (D. simulans)*	1.230	0.221	neutral evolution
	cuckoo (D. melanogaster)	-1.188	0.237	neutral evolution
woodpecker 11 (CG46513)	*LOC6725892 (D. simulans)*	1.131	0.260	neutral evolution
	cuckoo (D. melanogaster)	-0.898	0.371	neutral evolution
